# Colonel William Warren Potter, M.D.

**Published:** 1911-12

**Authors:** 


### OBITUARY

ELOQUENT tributes were paid to the memory of the late Colonel William Warren Potter, M.D., who conducted this JOURNAL so long and so ably, at the fall meeting of the American Association of Obstetricians and Gynecologists. Dr. Potter had been Secretary of this Association for many years. The memorials by Drs. McMurtry and Reed were especially touching, showing warm personal friendship as well as an appreciation of Dr. Potter's professional, literary and official services.

